# Supplementary material for: Complex‐centric proteome profiling by SEC‐SWATH‐MS
Source: Mol Syst Biol. 2019 Jan 14;15(1):e8438. doi: 10.15252/msb.20188438 (PMC6346213; doi:10.15252/msb.20188438)
Supplement: Supplementary file 8 — Dataset EV7 [file MSB-15-e8438-s008.zip › feature_plots_string/Q92750.pdf]

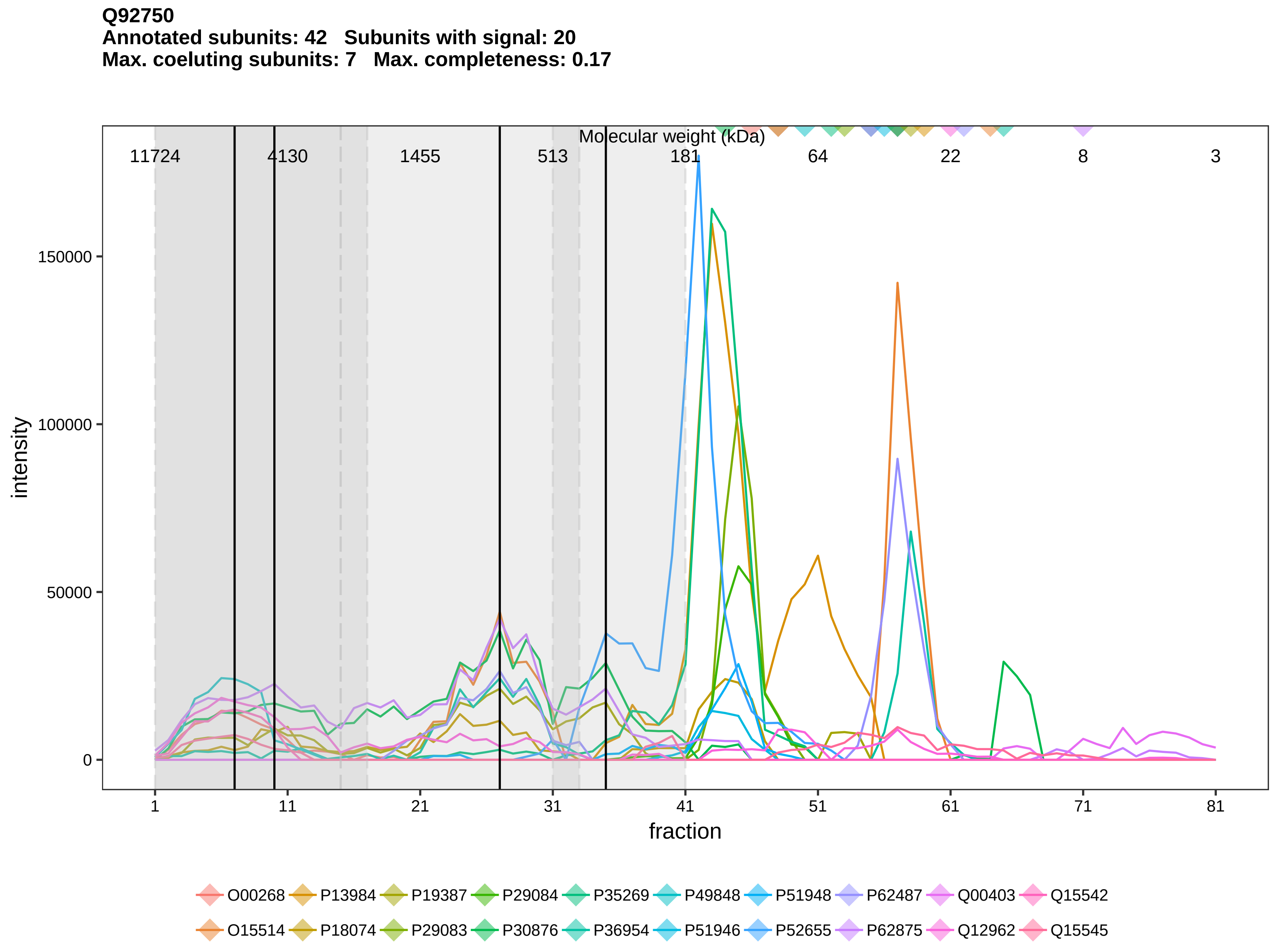
Q92750
Annotated subunits: 42 Subunits with signal: 20
Max. coeluting subunits: 7 Max. completeness: 0.17
Molecular weight (kDa)
11724
4130
1455
513
181
64
22
8
3
intensity
150000
100000
50000
0
1
11
21
31
41
51
61
71
81
fraction
O00268
P13984
P19387
P29084
P35269
P49848
P51948
P62487
Q00403
Q15542
O15514
P18074
P29083
P30876
P36954
P51946
P52655
P62875
Q12962
Q15545
